# BUB1 and SURVIVIN proteins are not degraded after a prolonged mitosis and accumulate in the nuclei of HCT116 cells

**DOI:** 10.1038/cddiscovery.2016.79

**Published:** 2016-10-24

**Authors:** Marco A Andonegui-Elguera, Rodrigo E Cáceres-Gutiérrez, Fernando Luna-Maldonado, Alejandro López-Saavedra, José Díaz-Chávez, Fernanda Cisneros-Soberanis, Diddier Prada, Julia Mendoza-Pérez, Luis A Herrera

**Affiliations:** 1 Unidad de Investigación Biomédica en Cáncer, Instituto de Investigaciones Biomédicas - Universidad Nacional Autónoma de México; Insituto Nacional de Cancerología, México City 14080, Mexico

## Abstract

Spindle poisons activate the spindle assembly checkpoint and prevent mitotic exit until cells die or override the arrest. Several studies have focused on spindle poison-mediated cell death, but less is known about consequences in cells that survive a mitotic arrest. During mitosis, proteins such as CYCLIN B, SECURIN, BUB1 and SURVIVIN are degraded in order to allow mitotic exit, and these proteins are maintained at low levels in the next interphase. In contrast, exit from a prolonged mitosis depends only on degradation of CYCLIN B; it is not known whether the levels of other proteins decrease or remain high. Here, we analyzed the levels and localization of the BUB1 and SURVIVIN proteins in cells that escaped from a paclitaxel-mediated prolonged mitosis. We compared cells with a short arrest (HCT116 cells) with cells that spent more time in mitosis (HT29 cells) after paclitaxel treatment. BUB1 and SURVIVIN were not degraded and remained localized to the nuclei of HCT116 cells after a mitotic arrest. Moreover, BUB1 nuclear foci were observed; BUB1 did not colocalize with centromere proteins. In HT29 cells, the levels of BUB1 and SURVIVIN decreased during the arrest, and these proteins were not present in cells that reached the next interphase. Using time-lapse imaging, we observed morphological heterogeneity in HCT116 cells that escaped from the arrest; this heterogeneity was due to the cytokinesis-like mechanism by which the cells exited mitosis. Thus, our results show that high levels of BUB1 and SURVIVIN can be maintained after a mitotic arrest, which may promote resistance to cell death.

## Introduction

Mitosis is an important target of cancer treatments. Drugs such as paclitaxel bind tubulin stabilize its polymerization, and activate the spindle assembly checkpoint (SAC), a surveillance mechanism that monitors the binding of microtubules to chromosomes.^1–3^ When this union is not achieved, the SAC prevents anaphase onset by inhibiting the anaphase-promoting complex/cyclosome (APC/C) until all chromosomes are attached to microtubules.^4^ In paclitaxel-treated cells, the arrest is maintained until cells escape from mitosis or die. It is widely believed that a prolonged mitosis is central to paclitaxel-induced cell death; however, the mechanistic details by which this drug kills cells remain unclear. Regardless of the specific mechanism of cell death after a stalled mitosis, BCL2 family proteins such as MCL1 are key factors that mediate the cell fate.^5–8^ Although several studies have focused on spindle poison-mediated cell death, less is known about consequences in cells that survive a mitotic arrest.

Several proteins are ubiquitylated by APC/C and members of the SCF family in order to be degraded by the proteasome.^9^ Ubiquitylation of CYCLIN B is mediated by the APC/C and its cofactor CDC20, and degradation of CYCLIN B is a requisite for mitotic exit via inhibition of CDK1 activity.^10^ Although APC/C is inhibited by the SAC during a protracted mitosis, some gradual degradation of CYCLIN B is achieved. When CYCLIN B levels drop below a certain threshold, CDK1 activity decreases, and cells escape from mitosis. However, this type of mitotic ‘slippage’ is not coordinated with typical degradation of other proteins at the end of mitosis. Indeed, the APC/C substrate TPX2 has been shown to be maintained at high levels after a mitotic slippage.^11^ Therefore, determining whether other proteins that are typically degraded at mitotic exit are maintained at high levels after a mitotic arrest is relevant, especially if their presence in G1 could affect cell behavior.

BUB1 and SURVIVIN are mitotic proteins that are degraded at the end of mitosis (both are ubiquitylated by APC/C bound to its cofactor CDH1) but that have roles in other processes in addition to mitosis. BUB1 is a mitotic kinase that recruits SAC proteins to kinetochores during prometaphase;^12,13^ it phosphorylates histone H2A at threonine 120, which is a mark for the recruitment of SGO1 (a protein that maintains sister chromatid cohesion in the centromere) and the chromosomal passenger complex (CPC);^14^ its kinase activity inhibits APC/C through the phosphorylation of CDC20.^12^ BUB1 and BUB3 have been proposed to regulate caspase-independent mitotic death in cells treated with spindle poisons.^15,16^ In addition to its functions in mitosis, BUB1 participates in the DNA damage response (DDR),^17^ and BUB1 kinase activity was recently shown to promote TGF-*β* receptor activation.^18^ In contrast, SURVIVIN is an inhibitor of apoptosis (IAP) protein that is a component of the CPC along with AURORA B, INCENP and BOREALIN.^19^ The CPC regulates chromosome-microtubule attachment, the mitotic checkpoint and cytokinesis in mitosis.^19^ Moreover, SURVIVIN acts as an antiapoptotic protein by the association and inhibition of SMAC/DIABLO and XIAP.^20^

We investigated the consequences of a mitotic arrest on the localization and levels of BUB1 and SURVIVIN. We compared two cell lines that behave differently when they are treated with the spindle poison paclitaxel. We followed the cell fate after a mitotic arrest as well as the levels and localization of BUB1 and SURVIVIN in cells that escaped from the arrest.

## Results

### Mitotic arrest is shorter in HCT116 cells than in HT29 cells

**Figure 1:**
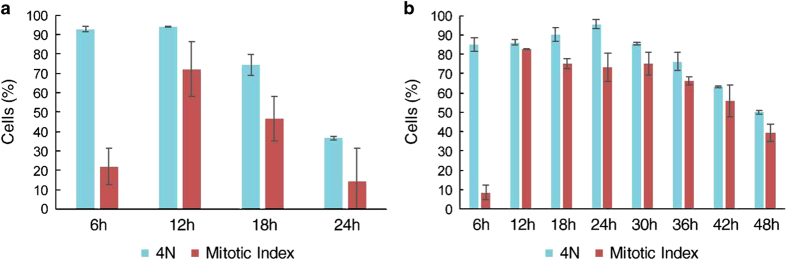
Different patterns of arrest and slippage in HCT116 and HT29 cell lines. Cells were synchronized by a thymidine block for 18 h. After the block, paclitaxel (100 nM) was added, and cells were collected at the indicated timepoints. (**a**) Most HCT116 cells escape from mitosis at 24 h after paclitaxel treatment. (**b**) In contrast, almost 40% of HT29 cells are still in mitosis after 48 h of paclitaxel treatment. The mitotic index was determined by light microscopy, whereas the 4N cell population was defined by the DNA content as determined by flow cytometry.

To determine the concentration of paclitaxel that can arrest most cells in mitosis, we performed a dose titration experiment in HCT116 and HT29 cells after cell synchronization by a thymidine block. Paclitaxel (100 nM) induced a protracted mitosis in most HCT116 and HT29 cells with similar efficiency compared to a higher paclitaxel concentration (Supplementary Figure 1). Therefore, this concentration was used in the next experiments with synchronized cells. We found a difference in the length of mitotic arrest between HCT116 and HT29 cells. Analysis of the mitotic index in HCT116 cells showed a peak at 12 h of treatment (Figure 1a) and a decrease at 18 h; approximately 10% of cells were in mitosis at 24 h (Figure 1a). We also measured the DNA content in HCT116 cells by flow cytometry and observed that 4N cells had a pattern similar to that of the mitotic index, but with a peak at 6 h, suggesting that some cells reached G2 phase but were not yet in mitosis (Figure 1a). In contrast, HT29 cells spend more time in mitosis than did HCT116 cells, with most cells in mitosis at 12 h and a decrease in the mitotic index after 36 h of treatment; 40% of cells were still in mitosis after 48 h of treatment (Figure 1b).

**Figure 2:**
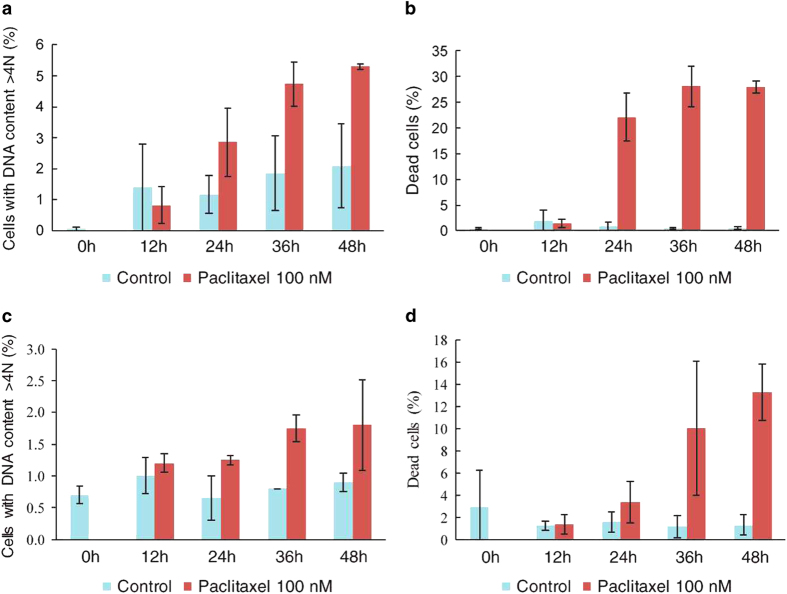
Consequences of mitotic arrest in HCT116 and HT29 cells. After the cells were synchronized by thymidine block (18 h), they were exposed to paclitaxel (100 nM). (**a**) Polyploid cells that escape from mitosis and enter a new cell cycle (DNA content>4) are evident after 24 h of treatment. (**b**) Simultaneously, the percentage of dead cells increases (DNA content<2N). (**c**) The number of polyploid HT29 cells is not significant after 48 h of treatment in accordance with longer arrest in this cell line. (**d**) After HT29 cells are treated with paclitaxel for 36 h, the percentage of dead cells increases but remains near 10% until 48 h.

Cells that escape from mitosis after an arrest can die in the next interphase or survive. We evaluated whether the cells that survive after a prolonged mitotic arrest enter in a new cycle of division by measuring their DNA content. In HCT116 cells, the percentage of polyploid cells with a DNA content>4N (cells that escaped from mitotic arrest and entered a new round of DNA replication) increased after 24 h of treatment with paclitaxel (Figure 2a); simultaneously, the fraction of dead cells increased (Figure 2b). In contrast, consistent with a longer mitotic arrest in HT29 cells, we did not observe cell death until 36 h of paclitaxel treatment, and the polyploid cell fraction that entered a new cell cycle did not increase considerably (Figures 2c and d). Therefore, HCT116 cells spend less time in mitosis in comparison with HT29 cells after paclitaxel treatment, and cells that escape from mitosis can enter a new cell cycle.

### BUB1 and SURVIVIN protein levels decrease with no relation to mitotic slippage

**Figure 3:**
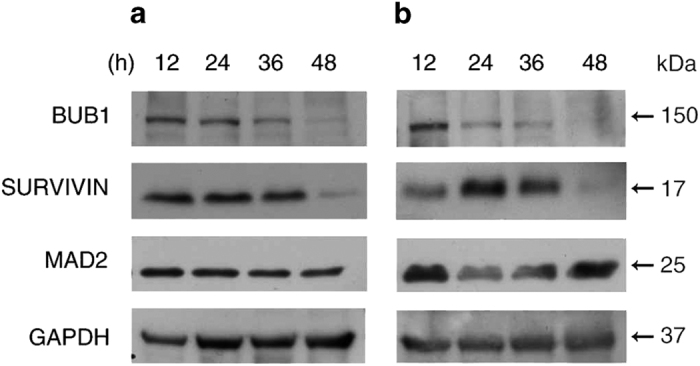
BUB1 and SURVIVIN protein levels decrease in a similar pattern during a prolonged mitosis in HCT116 and HT29 cells. Western blot analysis of paclitaxel-treated HCT116 (**a**) and HT29 (**b**) cells after thymidine block. In both HCT116 and HT29 cell lines, BUB1 and SURVIVIN levels diminish over time, whereas the levels of the mitotic checkpoint protein MAD2 remain constant during mitotic arrest.

We next compared BUB1 and SURVIVIN protein levels during mitotic arrest in HCT116 and HT29 cells. Western blot analysis of BUB1 and SURVIVIN in HCT116 cells showed a dramatic decrease in the levels of both proteins at 48 h after paclitaxel treatment (Figure 3a). BUB1 levels began to decrease at 36 h, with minimal levels at 48 h, while SURVIVIN levels remained uniform until 36 h and decreased at 48 h (Figure 3a). Interestingly, BUB1 and SURVIVIN levels did not change at 24 h of treatment, when most cells had already escaped from mitosis (Figures 1a and 3a). In HT29 cells, BUB1 levels began to decrease after 24 h of paclitaxel treatment and continued to decrease until 48 h, and SURVIVIN levels increased from 12 to 24 h and diminished at 48 h (Figure 3b). In HT29 cells, the levels of both proteins were markedly diminished at 48 h, although 50% of the cells were still in mitosis. Therefore, mitotic slippage does not coincide with degradation of these proteins in both cell lines. To determine whether these patterns were a general degradation mechanism of mitotic proteins, we evaluated the protein levels of MAD2, a protein whose levels do not change during the cell cycle. We did not observe changes in MAD2 protein levels during paclitaxel treatment (Figures 3a and b).

### BUB1 and SURVIVIN show nuclear localization after a prolonged mitosis in HCT116 cells

**Figure 4:**
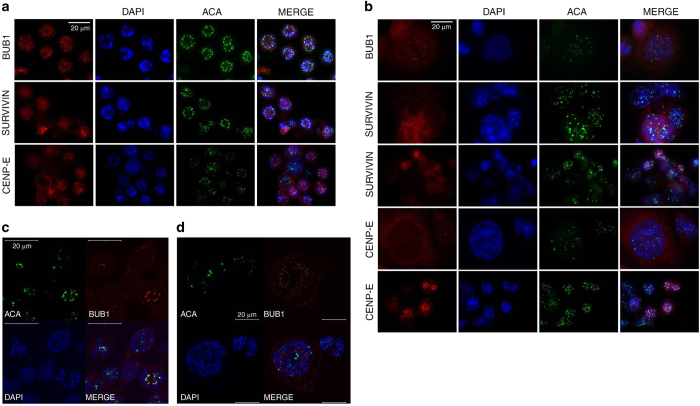
BUB1 and SURVIVIN showed nuclear localization after a mitotic arrest in HCT116 cells. (**a**) Representative images of immunostaining for BUB1, SURVIVIN and CENP-E showing kinetochore localization after 12 h of paclitaxel treatment. (**b**) After 24 h of paclitaxel treatment, cells that escaped from mitotic arrest showed nuclear foci localization of BUB1, nuclear localization of SURVIVIN without foci formation, and cytoplasmic localization of CENP-E. Localization of BUB1 in control (**c**) and 24 h paclitaxel-treated (**d**) HCT116 cells was determined by confocal microscopy. DAPI was used for DNA staining. Scale bar is equal to 20 *μ*m.

Because BUB1 and SURVIVIN are apparently still present in HCT116 cells that escaped from a prolonged mitosis at 24 h of treatment, we investigate whether both proteins maintained kinetochore localization in individual cells that achieved mitotic slippage. After HCT116 mitotic cells were treated with paclitaxel for 12 h, BUB1 and SURVIVIN were still present in the kinetochores of these cells (Figure 4a), although the BUB1 signal was weaker in these cells than in control cells (Supplementary Figure 2). Few mitotic cells were present at 24 h of paclitaxel treatment, and the morphology of interphase cells was variable (Figures 4b, d). At this time, cells in mitosis showed diffuse localization of BUB1. However, we found some larger interphase cells with BUB1 foci localized in their nuclei; these foci were not at the centromere region (Figures 4b and d). SURVIVIN was also present in the nuclei of these larger cells, although its signal was not concentrated in foci (Figure 4b). Moreover, the SURVIVIN signal was stronger in the few cells that were still in mitosis at 24 h of paclitaxel treatment (Figure 4b). The mitotic kinesin CENP-E, which is degraded in anaphase by a Skp1-dependent mechanism,^21^ was invariably localized at the kinetochores of HCT116 cells in mitosis and in the cytoplasm of larger cells that escaped from arrest (Figures 4a and b); thus, the localization of BUB1 and SURVIVIN is specific to these proteins.

**Figure 5:**
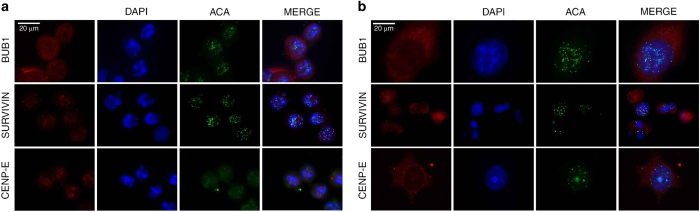
BUB1 and SURVIVIN are maintained in HT29 cell nuclei after a mitotic arrest. Representative images of immunostaining for BUB1, SURVIVIN and CENP-E after 12 h (**a**) or 48 h (**b**) of paclitaxel treatment in HT29 cells. DAPI was used for DNA staining. Scale bar is equal to 20 *μ*m.

In contrast, BUB1 showed diffuse localization in HT29 cells during mitotic arrest (Figures 5a and b) and was localized in the cytoplasm in cells that escaped from the delayed mitosis (Figure 5b). Moreover, SURVIVIN and CENP-E were associated with the chromosomes until 48 h of treatment in mitotic cells and were localized in the cytoplasm in post-mitotic cells (Figure 5b, Supplementary Figure 3). Therefore, in HCT116 cells (which showed a shorter mitotic arrest), BUB1 and SURVIVIN are not degraded at the mitotic exit, and they maintain their nuclear localization. Moreover, BUB1 foci are not co-localized with the centromere region.

### Variation in cell morphology after a prolonged mitosis is related to mitotic exit

**Figure 6:**
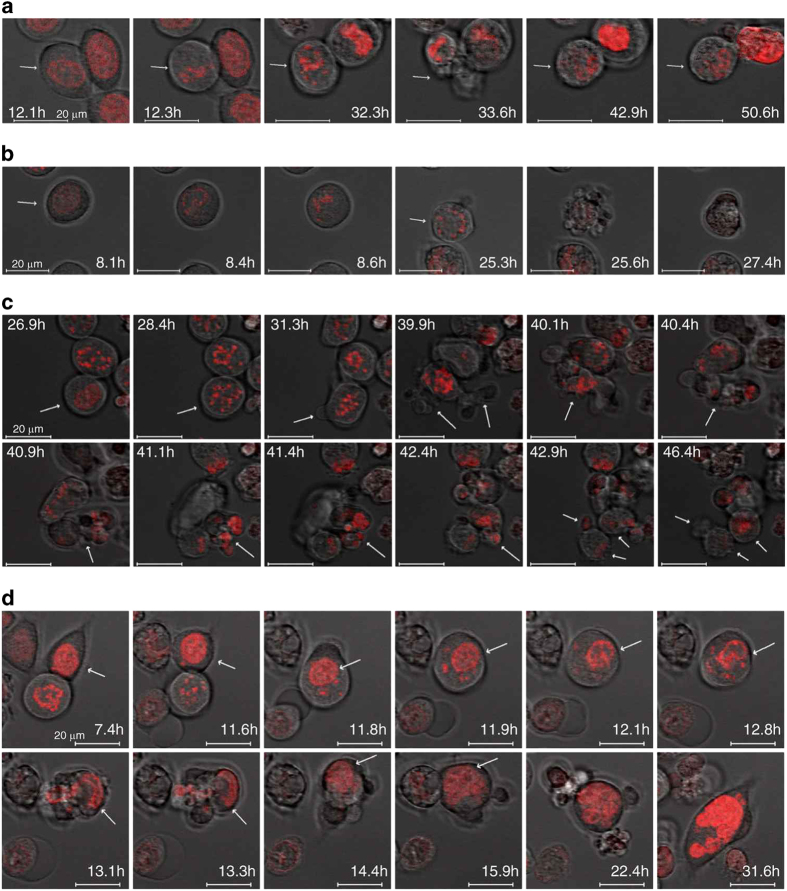
Heterogeneity in cell fate and morphology after a mitotic arrest. HCT116-H2BDsRed cells were synchronized by thymidine block, treated with paclitaxel (100 nM) and analyzed by time lapse. (**a**) Cell enters mitosis (arrow) at 12.3 h and exits mitosis at 33.6 h (cytokinesis-like). The cell is reorganized at 42.9 h and is maintained until the end of the time-lapse imaging (50.6 h). (**b**) Cell dying in mitosis (arrow). Chromosome condensation is evident at 25.3 h; at 25.6 h, membrane blebbing is initiated, and the cell dies. (**c**) Cell exits mitosis and divides into fragments (arrow). Cell enters mitosis at 28.4 h. Membrane is protruding at 31.3 h, and a cytokinesis-like state with membrane extensions is evident at 39.9 h (arrows). The cell is reorganized into three fragments (arrows) at 42.9 h. (**d**) Larger cell forms after mitotic arrest (arrow). Chromosome condensation begins at 12.1 h, chromosome decondensation begins at 13.1 h, and the cell is reformed into a larger cell at 31.6 h. Scale bar equal to 20 *μ*m.

We wanted to know whether the larger HCT116 cells with nuclear localization of BUB1 and SURVIVIN could have formed after paclitaxel-mediated mitotic arrest. To investigate this question, we followed the cell fate of HCT116-H2BDsRed cells by time-lapse microscopy during a prolonged mitosis (50 h). Mitotic arrest and slippage were similar to those in the experiments with fixed cells. However, the maximum arrest was observed between 20 and 25 h, and most cells were in interphase after 35 h of treatment (Supplementary Figure 4A). As reported previously, cell fate after mitotic arrest was heterogeneous (Figures 6a-d and Supplementary Figure 4). Cells entered mitosis with different kinetics; some of these cells died in mitosis (Figure 6b), others exited from mitosis and died in the next interphase (Supplementary Figure 4A), and some cells remained in interphase until the end of the experiment (Figures 6a, c and d). The outcome was not related to the time of entry into mitosis or the length of the mitotic arrest. Regarding cell morphology, we observed heterogeneity in the reorganization of cells after mitotic slippage. As cells entered mitosis, they became rounded, and their chromosomes became condensed. Before chromosome decondensation, cells that escape from mitosis showed dramatic changes in morphology (Figures 6a, c and d) similar to the beginning of cytokinesis. After these cytokinesis-like changes, most cells reorganized into an individual cell, but other cells achieved complete division and formed two or more fragments (Figure 6c). Moreover, we observed cells that reorganized in larger multinucleated cells similar to those fixed cells with nuclear localization of BUB1 and SURVIVIN (Figure 6d). Therefore, the morphology variation in HCT116 cells after a mitotic slippage may be due to the mechanism the cell uses to resolve the cytokinesis-like state at mitotic exit, and larger multinucleated cells are formed in this way.

## Discussion

We compared cell lines with different dynamics during a prolonged mitosis. Consistent with previous reports, in response to paclitaxel treatment, HT29 cells showed a longer arrest in mitosis than did HCT116 cells.^22–24^ It is known that APC/C-CDC20 has low activity during mitotic arrest and can ubiquitylate and promote degradation of CYCLIN B. If this degradation diminishes CDK1 activity under a certain threshold, mitotic slippage is achieved.^11,25^ In this scenario, it will be important to determine whether APC/C-CDC20 activity is higher in HCT116 cells compared with HT29 cells during mitotic arrest or whether the decreased level of CYCLIN B to overcome the arrest is lower in HCT116 cells, which would allow them to escape from mitosis faster than HT29 cells.

Interestingly, we found that regardless of the length of mitotic arrest, BUB1 and SURVIVIN protein levels declined at the same rate in both cell lines. In unperturbed mitosis, BUB1 and SURVIVIN are ubiquitylated by APC/C-CDH1 and degraded at mitotic exit.^26,27^ Because CDH1 is inhibited by CDK1-CYCLIN B phosphorylation,^28^ it is conceivable that targets of APC/C-CDH1 are not ubiquitylated during a protracted mitosis due to inhibition of CYCLIN B degradation. Indeed, we found that BUB1 and SURVIVIN are present in HCT116 cells that escaped mitotic arrest. However, BUB1 and SURVIVIN levels also diminished in HT29 cells, which are in mitosis longer than HCT116 cells are. Then, APC/C-CDH1 could have some remaining activity during a protracted mitosis, and if the arrest is long enough (e.g., HT29 cells), this activity would allow the degradation of APC/C-CDH1 targets such as BUB1 and SURVIVIN before slippage. In contrast, in cells with a shorter arrest (e.g., HCT116 cells), this activity would not diminish the levels of those targets, and the cells with higher levels of APC/C-CDH1 targets would escape from mitosis. Another mechanism leading to BUB1 and SURVIVIN degradation could be caspase-mediated: BUB1 is a known caspase target^29^ whereas SURVIVIN bears two putative caspase cleavage sites, according to *in silico* prediction (http://caspdb.sanfordburnham.org/index.php). Hence, it would be interesting to evaluate the levels of these proteins in individual, paclitaxel-treated cells under caspase inhibition.

Accordingly with this hypothesis, Brito and Rieder demonstrated that the protein TPX2 (a target of APC/C-CDH1) is present in the nuclei of RPE1 cells in interphase after nocodazole treatment. Consistent with this report, we found that BUB1 is maintained in nuclear foci in HCT116 cells after paclitaxel treatment.^11^ However, BUB1 nuclear foci are not localized on the centromeric region; therefore, this localization does not coincide with other kinetochore proteins. It is known that spindle poison-mediated mitotic arrest causes DNA damage and *γ*-H2AX accumulation.^30^ In contrast, BUB1 participates in the DDR during interphase.^17^ It will be interesting to evaluate whether BUB1 foci colocalize with sites of DNA damage and participates in the DNA repair system.

We also observed that SURVIVIN accumulated in the nuclei of larger cells but was not localized in specific foci. Although it has been demonstrated that nuclear localization of SURVIVIN is not a cytoprotective factor,^26^ increasing levels of SURVIVIN are related to antiapoptotic features and resistance to chemotherapeutic drugs. In addition, BUB1 is associated with negative regulation of cell death.^15,16^ Therefore, persistence of BUB1 and SURVIVIN in cells that override a mitotic arrest may promote mechanisms of resistance to cell death. Indeed, we observed that HCT116 cells that escaped from a mitotic arrest could enter a new DNA replication cycle.

We observed that the variable cell morphology observed after a prolonged mitosis is associated with mitotic exit. The division plane during cytokinesis is regulated by the spindle position and the microtubule organization in the central spindle. Moreover, formation of the contractile ring and the abscission during cytokinesis requires CDK-CYCLIN B inactivation.^31^ Microtubule stabilization by paclitaxel avoids central spindle formation, and gradual degradation of cyclin B could not be sufficient to achieve complete abscission in cells that reorganize into one cell. However, in cells where the level of Cyclin B degradation is lower, the division could be completed in aberrant planes, provoking the formation of cell fragments. Therefore, targeting mitosis and promoting cytokinesis could be a more efficient mechanism to kill cancer cells. Furthermore, spindle poisons that inhibit microtubule polymerization, such as nocodazole, could abolish actomyosin contractile ring organization. Thus, we hypothesize that these drugs induce a different phenotype from that caused by paclitaxel during mitotic exit.

Some factors that determine cell death after mitotic arrest have been described recently. However, little is known about cells that survive mitotic arrest. Our data suggest that the degradation mechanisms of mitotic proteins are not completely active during a mitotic arrest and that some proteins can be abnormally maintained in the next interphase in cells that override the arrest. However, it is not known whether these proteins may affect cell behavior in the next phases of the cell cycle. In contrast, the cytokinesis-like mechanism by which these cells exit mitosis may be responsible for the heterogeneous morphology of these cells after mitotic slippage, and this phenomenon could be a new target for spindle poison-treated cells.

## Materials and Methods

### Cell lines and culture conditions

HCT116 and HT29 (two cell lines established from human colon adenocarcinoma) cells were obtained from ATCC and cultured in McCoy’s 5A medium (GIBCO, Grand Island, NY, USA), supplemented with 10% fetal bovine serum (GIBCO) and 1% antibiotic-antimycotic (GIBCO) in a humidified atmosphere with 5% CO_2_ at 37 °C.

### Paclitaxel treatment

Paclitaxel (T7191, SIGMA-ALDRICH, St Louis, MO, USA) was prepared from a DMSO stock and was diluted using PBS-DMSO 1 : 1. The DMSO concentration was always less than 0.1%. Cells were synchronized by incubation with 2 mM thymidine for 18 h Then the cells were washed twice with PBS, and medium supplemented with paclitaxel or DMSO was added.

### Mitotic index

Determination of the mitotic index has been described elsewhere.^32^ Briefly, the cells were incubated in a hypotonic solution (HEPES-EGTA-KCl) for 20 min. Then, the cells were scraped, and 1 mL of 3 : 1 methanol/acetone was added. The cells were centrifuged and then incubated in 3 : 1 methanol/acetone solution at −20 °C for at least 30 min. Thereafter, the fixed cells were centrifuged and resuspended twice with methanol/acetone solution. The cells were placed on microscope slides for staining with methylene blue and eosin. At least 1000 cells were evaluated under a light microscope, and the percentage of mitotic cells was determined.

### Flow cytometric analysis

Cells were harvested and fixed in 70% cold ethanol at the indicated times after treatment. After the cells were fixed for at least 12 h at −20 °C, they were resuspended in PBS and centrifuged at 1500 r.p.m. Then, the cells were stained with 10 *μ*g/mL propidium iodide in 1.1% sodium citrate buffer supplemented with 0.25 mg/mL RNase A for 2 h. DNA content was measured using a FACSCanto II cytometer (Becton Dickinson, San Jose, CA, USA) with FACSDIVA 6.1.3 software (Becton Dickinson). The subG1 and >4N cell populations were estimated by analyzing the DNA content distribution using FACSDiva software. Cell cycle phases were determined using MODFit LT 4.0 software (Becton Dickinson).

### Western blot analysis

Cells were grown in 10 cm dishes and then incubated for 5 min with Cell Lysis Buffer (#9803, Cell Signaling, Danvers, MA, USA) supplemented with 100 *μ*M PMSF. The cells were scraped and collected in tubes, and cell lysates were centrifuged for 20 min at 14 000 r.p.m. Proteins samples were resolved on 10 or 12% Tris-glycine gels and transferred onto PVDF membranes. Then, the membranes were incubated with anti-BUB1 (1 : 400; NBP1-88518, Novus, Littleton, CO, USA), anti-SURVIVIN (1 : 500; sc-17779, Santa Cruz, Dallas, TX, USA), anti-MAD2 (sc-47747, Santa Cruz) and anti-GAPDH (sc-25778, Santa Cruz). After the membranes were washed with TBS, they were incubated with peroxidase-conjugated goat anti-rabbit antibody (1 : 20 000; NB7187, Novus) and rabbit anti-mouse antibody (1 : 15 000; 61-6520, Sigma, St Louis, MO, USA) followed by chemiluminescent staining (WBKLS0100, Millipore, Billerica, MA, USA).

### Immunofluorescence

Cells were grown on cover slides in six-well culture flasks. Prior to fixation, the cells were rinsed in PBS and then fixed for 10 min with cold methanol and 1 min with cold acetone. Thereafter, coverslips were blocked in PBS containing 1% albumin for 1 h at room temperature before being processed for immunofluorescence. The following primary antibodies were used: anti-BUB1 (MAB3610, Millipore, 1 : 10), anti-Centromere FITC-conjugate (15-235-F, Antibodies Incorporated, Davis, CA, USA, 1 : 100), anti-CENP-E (sc-22790, Santa Cruz, 1 : 50), and anti-SURVIVIN (ab469, Abcam, Cambridge, MA, USA, 1 : 100). The following secondary antibodies were used: anti-mouse Cy3-conjugate (AP124C, Millipore, Temecula, CA, USA, 1 : 200), anti-rabbit Cy3 conjugate (AP132C, Millipore, Temecula, CA, USA, 1 : 200). DNA was counterstained with DAPI. Images were acquired on an AxioImager A1 upright microscope (Carl Ziess, Gottingen, Germany).

### Time-lapse microscopy

HCT116 cells stably expressing H2B-DsRed were seeded in a POC-R2 chamber (PECON, Erbach, Germany). The cells were synchronized by a thymidine block for 18 h, and then the cells were washed twice in PBS and incubated with paclitaxel. Imaging was performed using a LSM710-DUO confocal microscope (Carl Zeiss, Jena, Germany), with images collected every 8 min in three different planes during a 50 h period. Individual cells were viewed in ZEN 2011 software (Carl Zeiss) and analyzed manually.

## Abbreviations

APC/C: Anaphase Promoting Complex/Cyclosome
CPC: Chromosomal Passenger Complex
DDR: DNA Damage Response
SAC: Spindle Assembly Checkpoint.
