# Supplementary figures and images for: BUB1 and SURVIVIN proteins are not degraded after a prolonged mitosis and accumulate in the nuclei of HCT116 cells
Source: Cell Death Discov. 2016 Oct 24;2:16079–. doi: 10.1038/cddiscovery.2016.79 (PMC5081682; doi:10.1038/cddiscovery.2016.79)

**A**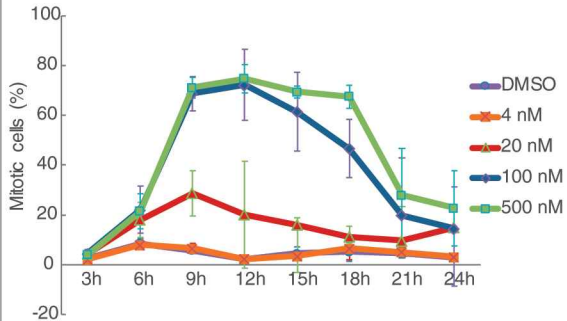**B**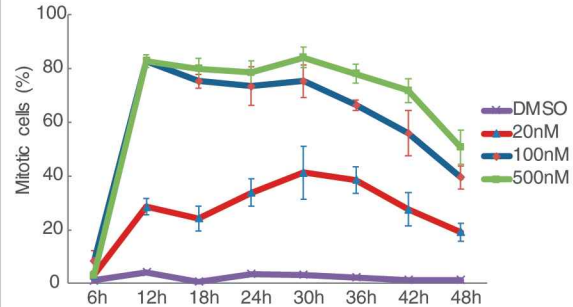

Supplement: Supplementary Figure 1 [file cddiscovery201679-s1.pdf]

**A**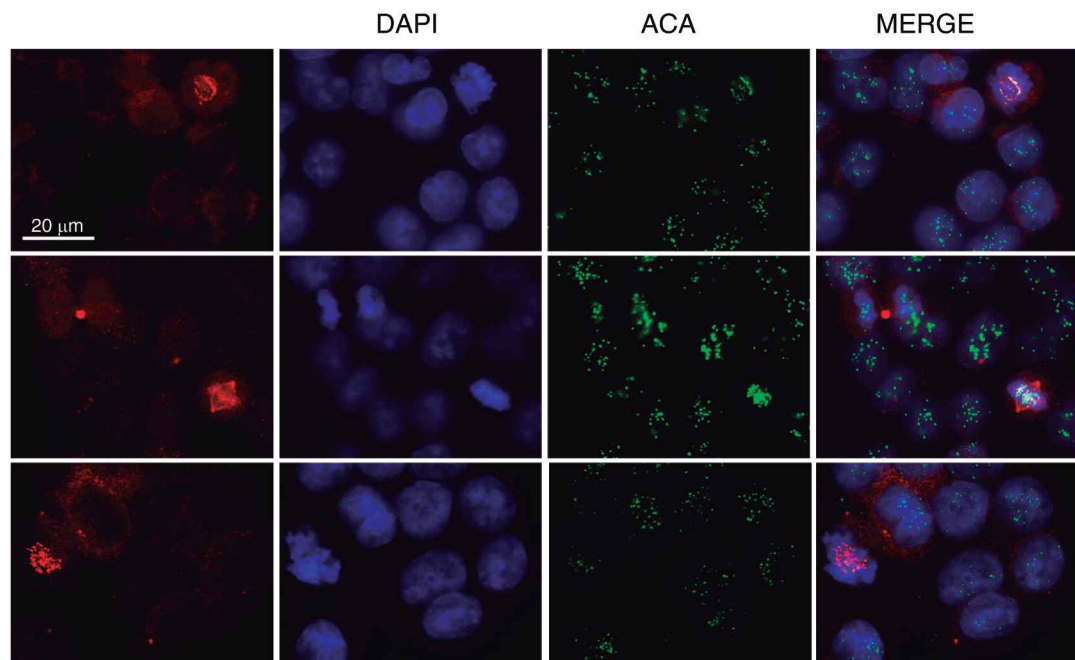**B**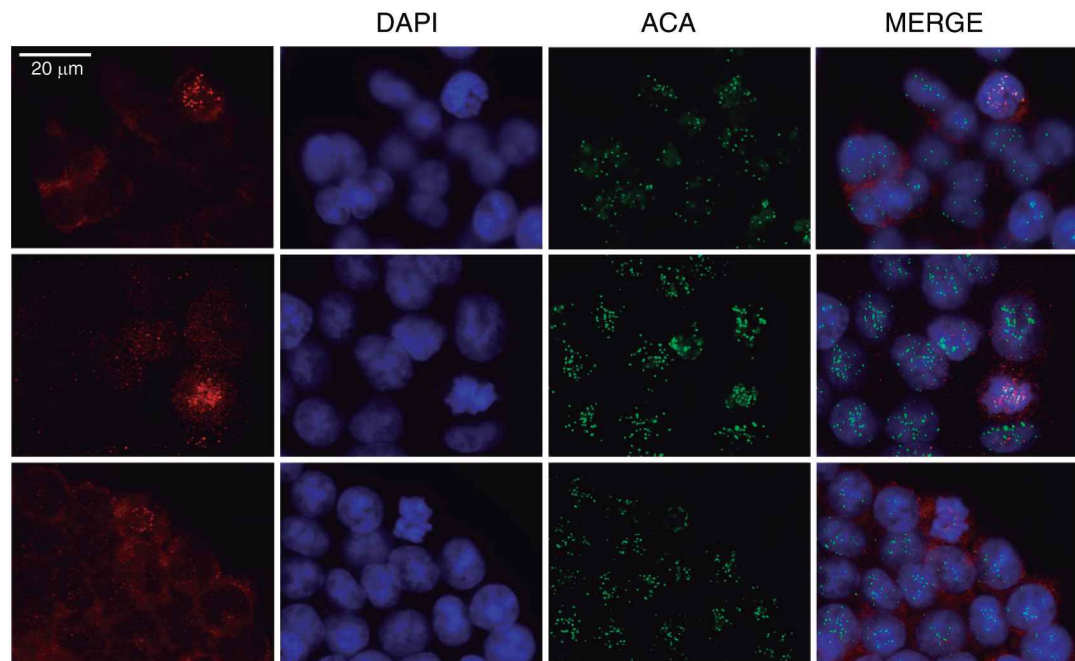

Supplement: Supplementary Figure 2 [file cddiscovery201679-s2.pdf]

**A**

DAPI

ACA

MERGE

BUB1

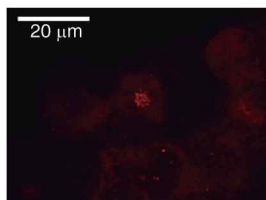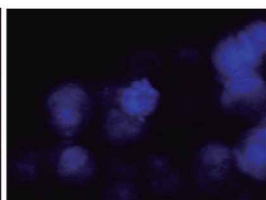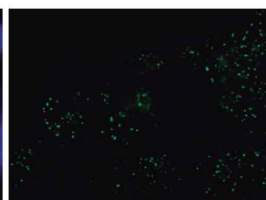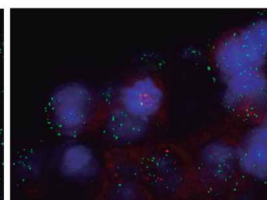

SURVIVIN

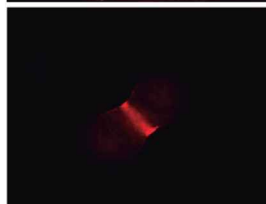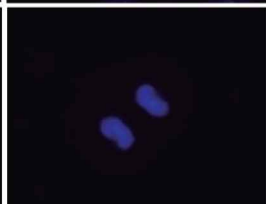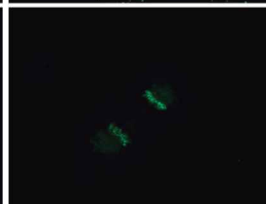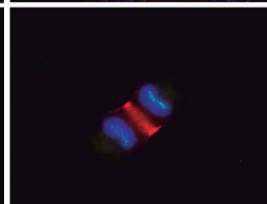

CENP-E

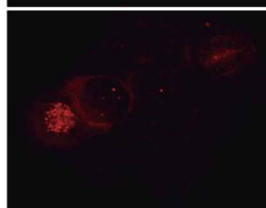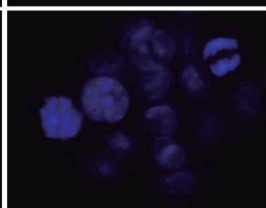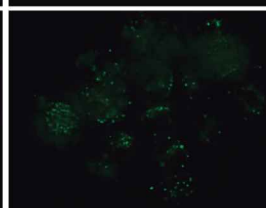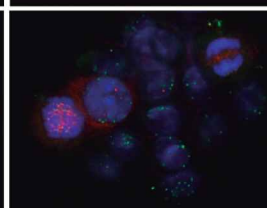**B**

DAPI

ACA

MERGE

BUB1

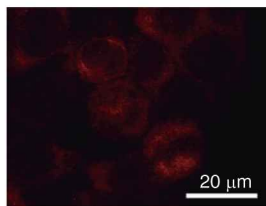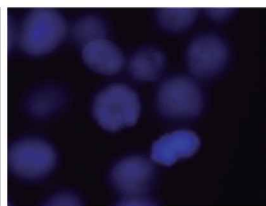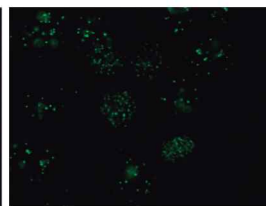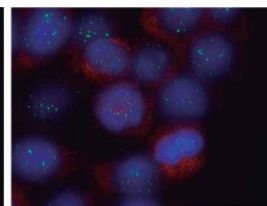

SURVIVIN

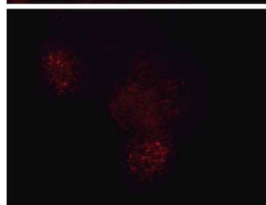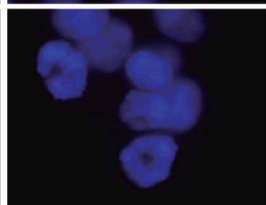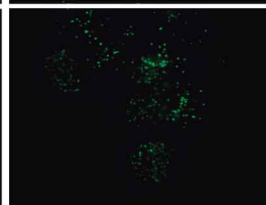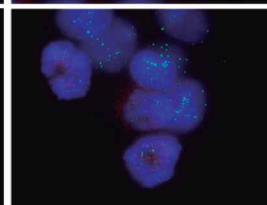

CENP-E

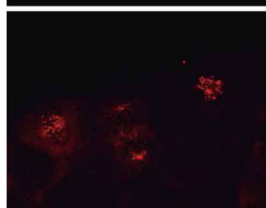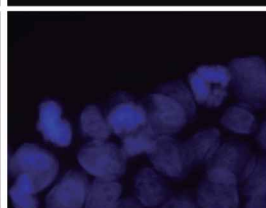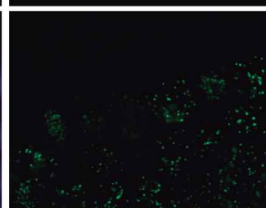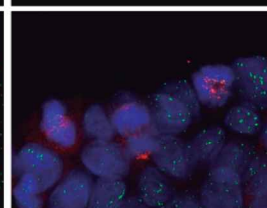

Supplement: Supplementary Figure 3 [file cddiscovery201679-s3.pdf]

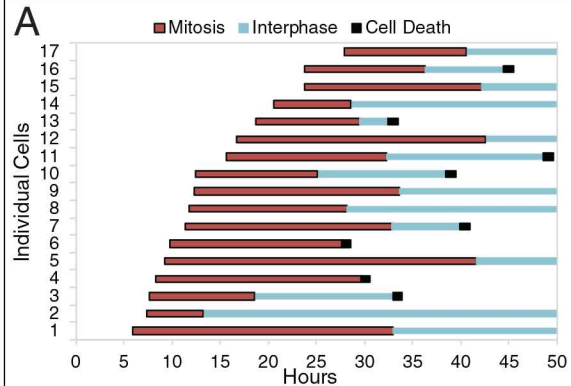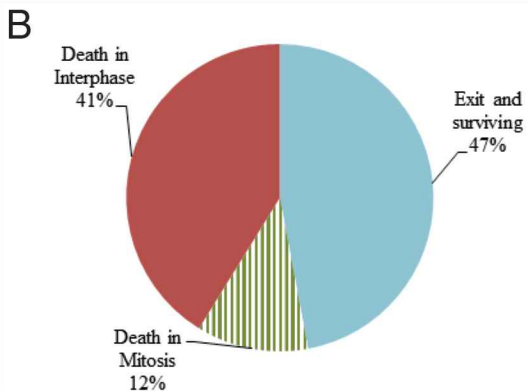

Supplement: Supplementary Figure 4 [file cddiscovery201679-s4.pdf]
